# A Natural Novel Mutation in the *bla*_NDM-5_ Promoter Reducing Carbapenems Resistance in a Clinical Escherichia coli Strain

**DOI:** 10.1128/spectrum.01183-21

**Published:** 2022-02-09

**Authors:** Yiming Li, Rong Zhang, Shaolin Wang

**Affiliations:** a College of Veterinary Medicine, China Agricultural University, Beijing, China; b The Second Affiliated Hospital of Zhejiang University, Zhejiang University, Hangzhou, China; INTHERES

**Keywords:** carbapenem-resistant, NDM-5, promoter mutation, *Escherichia coli*

## LETTER

The worldwide emergence of New Delhi metallo-β-lactamase (NDM) is of great concern. NDM could hydrolyze almost all β-lactam antibiotics, compromising antimicrobial treatments for patients with severe infection (1). Few NDM inhibitors are effective for treating infection caused by carbapenem-resistant bacteria, and no promising compounds have been developed in the process as future NDM inhibitors (2). To the best of our knowledge, no report of a naturally inactivated *bla*_NDM_ gene has been described so far. In this study, we identified a novel mutation in the promoter of IncX3-plasmid-carrying *bla*_NDM-5_ that leads to the reduction of carbapenems resistance in a clinical Escherichia coli strain.

The E. coli isolate Z503 was cultured from a fecal sample of a female patient in Zhejiang in 2018, using enrichment media supplemented with meropenem (0.25 μg/mL), and further confirmed by 16S rRNA gene sequencing. The presence of gene *bla*_NDM-5_ was screened by PCR, as described previously (3). NDM-producing Escherichia coli strains are usually associated with medium to high MIC for carbapenems. Interestingly, strain Z503 shows low resistance to meropenem (MIC = 1 μg/mL) and imipenem (MIC = 2 μg/mL), with MICs that remained within the susceptibility range. Whole-genome sequencing (WGS) of Z503 was performed using Illumina Hiseq2500 platform (Bionova) and Nanopore MinION sequencing platform (Oxford Nanopore Technologies). The whole-genome sequencing data were analyzed using multiple programs, including Unicycler (assembly), Prokka (annotation), and SRST2 (antibiotic resistance gene analysis) (3–5).

WGS revealed that E. coli Z503 harbored a 4.27-Mb circular chromosome and four plasmids, including IncX3 (∼46 kb), IncFIB (∼47 kb), IncFII (∼83 kb), and colRNA (∼5 kb) type plasmids. The *bla*_NDM-5_ gene was located on an IncX3 type plasmid, namely, p503-IncX3 (∼46 kb), which showed 98.61% identity to plasmid p3R-IncX3 (a *bla*_NDM-5_-carrying IncX3-plasmid identified in our previous study) (accession number CP049352). In addition to *bla*_NDM-5_, *bla*_TEM-98_ (extended-spectrum β-lactamase [ESBL]-encoding gene), *ampC1* and *mrdA* (β-lactam resistance genes), and 8 other antimicrobial resistant genes were identified in E. coli Z503. To investigate the conjugation efficiency of the p503-IncX3 plasmid, we mixed donor stain Z503 and recipient J53 at a ratio of 1:3 on LB agar and cultured them overnight. The mixtures were plated on LB agar containing meropenem (0.25 μg/mL) and sodium azide (150 μg/mL). The recombinant strains were confirmed by PCR-based screening. The plasmid p503-IncX3 could be successfully transferred at a high frequency (2.1 ± 3.0) × 10^−1^, and the meropenem MIC of transconjugants was only 0.5 μg/mL.

**FIG 1:**
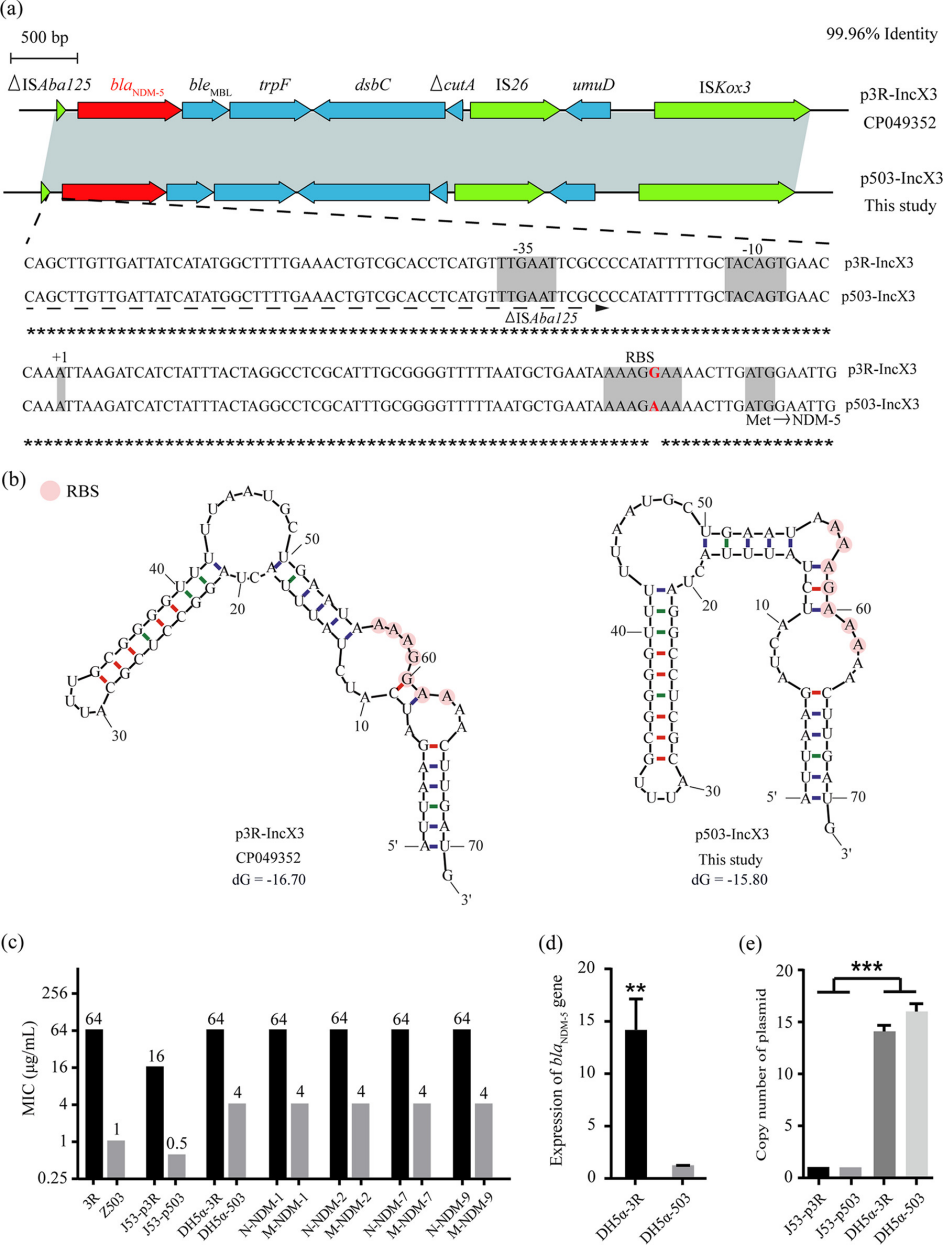
(a) Schematic structures of the flanking regions of the *bla*_NDM-5_ genes in plasmids p503-IncX3 and p3R-IncX3 (accession number CP049352). (b) The mRNA secondary structures prediction of the upstream sequence (68 bp) of the *bla*_NDM-5_ translational start codon. dG: loop free-energy for folding. In theory, the lower the loop free-energy is, the stabler a structure will form. (c) Meropenem MICs of the wild and constructed strains. 3R was an E. coli isolate harboring an IncX3-plasmid with a wild promoter upstream *bla*_NDM-5_, which was identified in a previous study (accession number CP049352). J53-3R and J53-503 were J53 strains containing p3R-IncX3 and p503-IncX3, respectively. DH5α-3R and N-NDM-1/2/7/9 were DH5α strains containing recombinant plasmids pET28a harboring *bla*_NDM_ and its wild promoters. DH5α-503 and M-NDM-1/2/7/9 were DH5α strains containing recombinant plasmids pET28a harboring *bla*_NDM_ and its mutational promoters. (d) Relative mRNA expression levels of *bla*_NDM-5_ genes in constructed strains as identified by quantitative reverse transcriptase PCR (qRT-PCR). The data are represented as 2^−ΔΔCT^ values. **, *P* < 0.01; ***, *P* < 0.001 (compared with DH5α-503). Each value indicates the mean ± standard deviation (SD) (*n* = 3). (e) Copy number of *bla*_NDM-5_ harboring plasmids in different strains.

The *bla*_NDM_-containing sequences identified share two common features. The upstream of *bla*_NDM_ is always the insertion sequence ISA*ba125*, while the downstream is always the bleomycin-resistant gene *ble*_MBL_ (1). The insertion sequence ISA*ba125* plays a key role in the mobilization of NDM (6, 7). A group of genes were located downstream of *ble*_MBL_, including *trpF*, *dsbC*, and *cutA* (1). Similar genetic context was found in E. coli Z503 in this study, with 99.96% homology to the corresponding sequences in plasmid p3R-IncX3 and its similar variants, which has been widely reported worldwide (Fig. 1a). Notably, a single base substitution (G to A) was observed in the promoter region of *bla*_NDM-5_ in plasmid p503-IncX3, altering the sequence of the ribosome-binding site (RBS) reported previously (Fig. 1a) (8).

To date, no report of silenced *bla*_NDM_ gene caused by natural promoter mutation has been described. In order to investigate variations of the promoter sequences of *bla*_NDM_, we downloaded 533 whole genomes of NDM-positive strains of different countries between 2015 and 2021 from the NCBI database. Only 3 strains were found to have a single base substitution (CP049352.1: g.5561A>G or g.5570C>T) in the promoter regions, suggesting the highly conserved sequence of the *bla*_NDM_ promoters. However, carbapenems resistance was still observed in all three strains, and none of the three mutations was present in the RBS.

The 5′ untranslated region (5′ UTR) of mRNA plays key roles in posttranscriptional regulatory mechanisms (9). If RBS sequences are located in a stable hairpin structure, the RBS-ribosome binding will be greatly affected. We predicted mRNA secondary structures of *bla*_NDM-5_ promoters as described previously (10). Compared to that in p3R-IncX3, the mRNA secondary structure of *bla*_NDM-5_ promoter in p503-IncX3 was more stable (Fig. 1b), which may be responsible for maintaining the susceptibility to carbapenems in E. coli Z503. To further understand the role of mutation in RBS, we cloned *bla*_NDM-5_ and its natural promoter sequence from p503-IncX3 and p3R-IncX3 into the pET28a expression vectors, respectively, and then transformed them into E. coli DH5α using the heat shock method. Our results indicated that the expression of NDM was significantly lower and the MIC of meropenem decreased 16 times (64 μg/mL to 4 μg/mL) after the base substitution at RBS (Fig. 1c and d).

Furthermore, *bla*_NDM_ genes other than *bla*_NDM-5_ (V88L, M154L), including *bla*_NDM-1_, *bla*_NDM-2_ (P28A), *bla*_NDM-7_ (D130N, M154L), and *bla*_NDM-9_ (E152K), with natural and mutated promoters were cloned into plasmid pET28a to explore the role of RBS mutation in NDM-1 variants. The *bla*_NDM_ genes included in this study covered the mutation sites shared by most NDM variants (Fig. S1), and most of them were also prevalent variants according to previous studies (3, 11). The same results (64 μg/mL to 4 μg/mL) were observed in all these NDM-1 variants (data not shown). The resistance phenotype (4 μg/mL) of DH5α-503 could be attributed to the higher copy numbers of the plasmid pET28a (Fig. 1e).

A better understanding of the structure-function relation of NDM enzymes is needed to facilitate the development of specific inhibitors. It was reported that amino acid substitutions in NDM-1 can significantly alter hydrolysis activity of enzymes (12, 13). Ali, Gupta, and Khan reported that non-active-site laboratory mutants of NDM-1 exhibited a 4-fold reduction in the MIC values with meropenem compared to those of the wild type (14). In our study, a single base substitution in the promoter of *bla*_NDM_ resulted in a 16-fold decrease of meropenem MIC.

In conclusion, this study reported that a natural novel mutation in the RBS sequence upstream of the *bla*_NDM-5_ gene was found to contribute to a very low level of NDM-5 expression and to further maintain the carbapenems susceptibility. Our findings indicated that inhibition of promoter activity may be a way to combat NDM-mediated carbapenems resistance in future studies.

### Data availability.

Whole-genome sequencing data that support the findings of this study have been deposited in the NCBI database under BioProject accession number PRJNA791555. Extra data supporting the findings of this study are available from the corresponding author upon reasonable request.
