# Supplementary material for: A Natural Novel Mutation in the blaNDM-5 Promoter Reducing Carbapenems Resistance in a Clinical Escherichia coli Strain
Source: Microbiol Spectr. 2022 Feb 9;10(1):e01183-21. doi: 10.1128/spectrum.01183-21 (PMC8826935; doi:10.1128/spectrum.01183-21)
Supplement: SUPPLEMENTAL FILE 1 — Supplemental material. Download Spectrum01183-21_Supplementary_materials_revised.pdf, PDF file, 0.3 MB [file spectrum01183-21_supplementary_materials_revised.pdf]

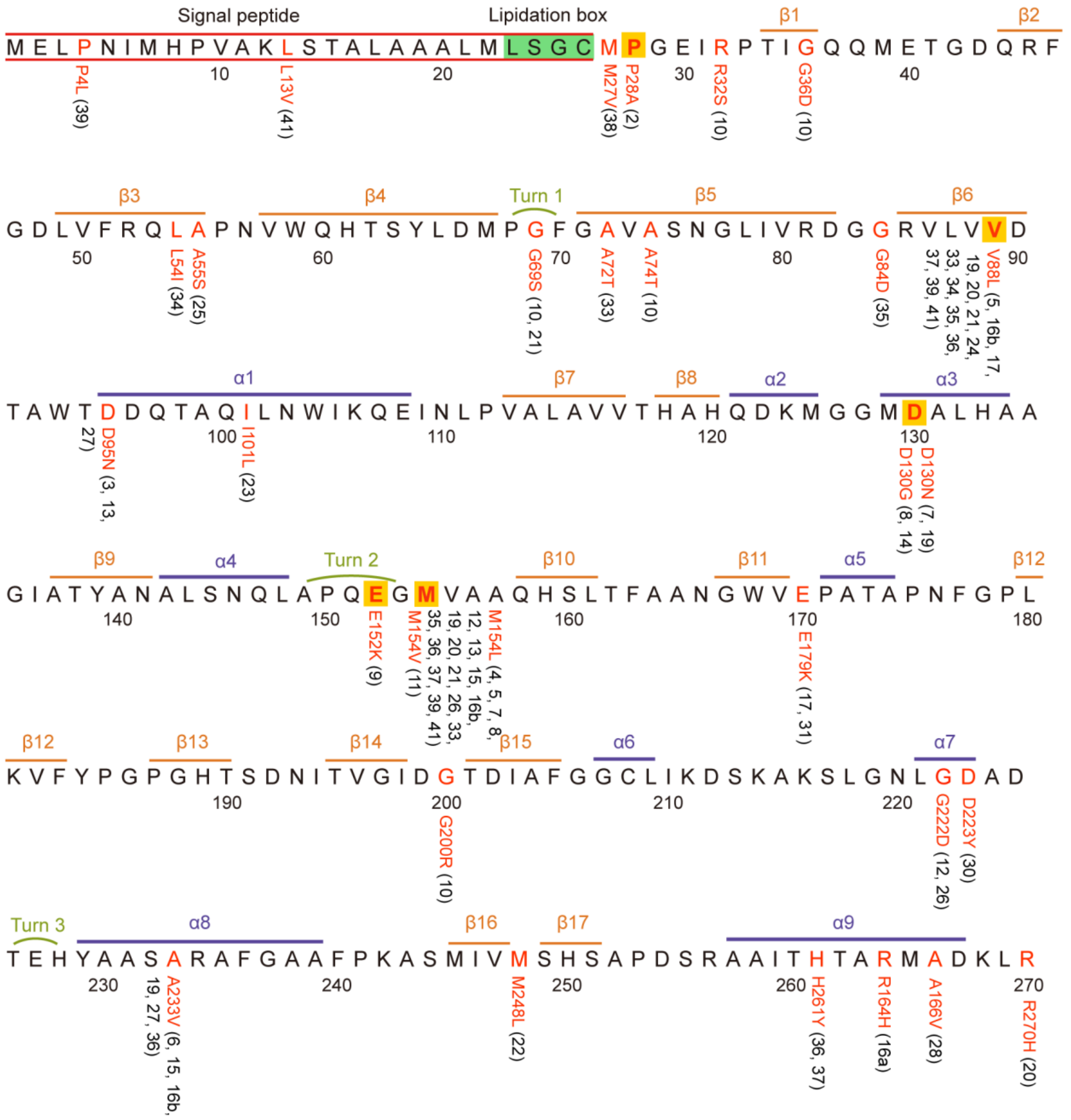

Figure S1. NDM amino acid sequence and NDM variants. The annotation of the NDM amino acid sequence is adopted from data reported under UniProt Accession no. C7C422 [1]. Amino acid substitutions compared with NDM-1 are highlighted in red with boldface type, and amino acid sites included in this study are highlighted in orange.

References

1. Wu W, Feng Y, Tang G, Qiao F, McNally A, Zong Z. 2019. NDM Metallo-β-Lactamases and Their Bacterial Producers in Health Care Settings. Clin Microbiol Rev 32:e00115-18.
